# Nicotinamide reverses deficits in puberty-born neurons and cognitive function after maternal separation

**DOI:** 10.1186/s12974-022-02591-y

**Published:** 2022-09-21

**Authors:** Keke Hao, Huiling Wang, Yuejin Zhang, Xinhui Xie, Huan Huang, Cheng Chen, Shilin Xu, Rui Xu, Chang Shu, Zhongchun Liu, Yuan Zhou, Gavin P. Reynolds, Gaohua Wang

**Affiliations:** 1 Department of Psychiatry, Renmin Hospital of Wuhan University, 238 Jiefang Road, Wuhan, 430060 China; 2 Hubei Institute of Neurology and Psychiatry Research, Wuhan, 430060 China; 3 Department of Psychiatry, Zhongxiang Hospital of Renmin Hospital of Wuhan University, Zhongxiang, 431900 China; 4 Hubei Provincial Key Laboratory of Developmentally Originated Disease, Wuhan, 430071 China; 5 Department of Physiology, School of Basic Medicine, Tongji Medical College, Huazhong University of Science and Technology, Wuhan, 430060 China; 6 Institute of Psychology, Chinese Academy of Sciences, Beijing, 100101 China; 7 Biomolecular Sciences Research Centre, Sheffield Hallam University, Sheffield, UK

**Keywords:** Schizophrenia, Early life stress, Maternal separation, Cognitive impairment, Neuroinflammation, Neuronal loss, Nicotinamide, Sirt3

## Abstract

**Background:**

Early life stress (ELS) is associated with the development of schizophrenia later in life. The hippocampus develops significantly during childhood and is extremely reactive to stress. In rodent models, ELS can induce neuroinflammation, hippocampal neuronal loss, and schizophrenia-like behavior. While nicotinamide (NAM) can inhibit microglial inflammation, it is unknown whether NAM treatment during adolescence reduces hippocampal neuronal loss and abnormal behaviors induced by ELS.

**Methods:**

Twenty-four hours of maternal separation (MS) of Wistar rat pups on post-natal day (PND)9 was used as an ELS. On PND35, animals received a single intraperitoneal injection of BrdU to label dividing neurons and were given NAM from PND35 to PND65. Behavioral testing was performed. Western blotting and immunofluorescence staining were used to detect nicotinamide adenine dinucleotide (NAD^+^)/Sirtuin3 (Sirt3)/superoxide dismutase 2 (SOD2) pathway-related proteins.

**Results:**

Compared with controls, only MS animals in the adult stage (PND56–65) but not the adolescent stage (PND31–40) exhibited pre-pulse inhibition deficits and cognitive impairments mimicking schizophrenia symptoms. MS decreased the survival and activity of puberty-born neurons and hippocampal NAD^+^ and Sirt3 expression in adulthood. These observations were related to an increase in acetylated SOD2, microglial activation, and significant increases in pro-inflammatory IL-1β, TNF-α, and IL-6 expression. All the effects of MS at PND9 were reversed by administering NAM in adolescence (PND35–65).

**Conclusions:**

MS may lead to schizophrenia-like phenotypes and persistent hippocampal abnormalities. NAM may be a safe and effective treatment in adolescence to restore normal hippocampal function and prevent or ameliorate schizophrenia-like behavior.

**Supplementary Information:**

The online version contains supplementary material available at 10.1186/s12974-022-02591-y.

## Introduction

Schizophrenia is a devastating psychiatric disorder caused by complex genetic and environmental factors. There are very strong epidemiological data showing that early life stresses (ELS) such as abuse and neglect are associated with higher rates of schizophrenia in later life [50]. Mother–infant interactions may significantly influence brain maturation, and stress caused by maternal separation (MS) may induce psychosis or susceptibility to psychotic diseases including schizophrenia. This association is borne out by experimental studies: the abnormal behaviors and molecular changes taking place after a single 24-h period of MS on post-natal day (PND) 9 in rats successfully recapitulate several features of human schizophrenia [23, 53, 60]. Therefore, the MS rat model has become a powerful tool for exploring the neurobiological basis of schizophrenia.

Neuronal loss is an important pathological feature of schizophrenia, and ELS may induce neuronal loss. The hippocampus is a critical area of the brain involved in stress responses, and it is enriched with corticosteroid receptors, particularly in dentate granule (DG) cells [19]. As the hippocampus also undergoes significant maturation during childhood and adolescence, this region is thought to be particularly vulnerable to the effects of ELS [45]. Previous neuropathological studies have observed decreased hippocampal neurogenesis and dentate gyrus volume in schizophrenia patients [2, 6]. The hippocampus is involved in cognitive function, and its dysfunction is thought to be related to the cognitive impairment seen in schizophrenia [1, 61, 66, 72]. Furthermore, meta-analyses have reported significant associations between childhood adversity and reduced hippocampal volume and impaired hippocampal function [13, 42]. Rodent MS models have demonstrated that ELS induces abnormal behaviors reminiscent of schizophrenia in adults by affecting hippocampal neurogenesis [7, 9–11]. However, the underlying molecular and pathological mechanisms driving these hippocampal changes have not been fully elucidated.

A neuroinflammatory state may contribute to the pathogenesis of many mental disorders including schizophrenia. Microglia are the main immune effector cells in the brain [17], and their function is also essential for central nervous system (CNS) development including neurogenesis, synaptic maturation, axonal growth, and myelination [59]. Normal microglia can release neurotrophic factors that promote neuron growth and migration [14, 49, 56]. However, over-activated microglia can induce neuronal damage and loss, enhancing the release of pro-inflammatory signals and triggering a positive feedback loop that further activates microglia and exacerbates inflammatory neuronal damage [24, 26]. It is hypothesized that this microglial hyperactivation, especially during critical developmental phases of the brain, can lead to structural and behavioral alterations that persist into adulthood [20, 36, 54]. We therefore hypothesized that microglial activation following ELS plays a critical role in the development of schizophrenia-like behaviors.

Nicotinamide adenine dinucleotide (NAD^+^) is an essential cofactor in multiple cellular metabolic reactions, and high NAD^+^ levels activate proteins involved in mitochondria quality control, such as Sirtuin3 (Sirt3). Sirt3 reinforces mitochondrial antioxidant defense responses by deacetylating and increasing superoxide dismutase 2 (SOD2) activity [51, 57]. A recent study suggested that mitochondrial NAD^+^ and Sirt3 activation prevents metabolic reprogramming of macrophages and restrains their pro-inflammatory phenotype [41]. NAD^+^ precursors have also been suggested as treatment adjuncts for schizophrenia [70]. One such precursor is nicotinamide (NAM), from which NAD^+^ is produced via the NAD^+^ salvage pathway. NAM is thought to protect neuron viability and brain function [55] and has been studied extensively in adult rats. However, there is little information about the neuroprotective function of NAM in the developing brain.

The primary aim of this study was to investigate whether administration of NAM during adolescence could rescue MS-induced neuronal loss and cognitive impairment in adult offspring. We also investigated the effects of NAM administration on microglial inflammation, NAD^+^ expression, Sirt3 activity, and oxidative stress parameters to determine the possible underlying mechanism of action.

## Materials and methods

### Animals and maternal separation

Twenty nulliparous female and 20 male 8-week-old Wistar rats were obtained from Beijing Vital Rival Laboratory Animal Technology Co., Ltd. (Beijing, China). Three or four rats of the same sex were caged together. Animals were mated at 3 months of age and the males removed one week later. Mated female rats were housed individually in ventilated plastic cages in a temperature- and humidity-controlled (22 ± 20 °C, 50 ± 10%) holding facility with a constant 12-h day–night cycle (lights, 08:00–20:00). All animals had free access to food and tap water.

MS was performed according to a previous protocol [53]. Females were checked twice daily for delivery (08:00 and 17:00), and the day of delivery was considered post-natal day (PND)0. Each pregnant rat provided on average 10 ± 2 offspring. On PND9, about ten litters of rats were randomly subjected to MS for 24 h. In brief, mothers were removed at 10:00. The pups remained in their home cages at room temperature for 24 h. On PND10, the mothers were placed back in their cages. The control groups grew naturally to adulthood. All the litters were otherwise left undisturbed except for routine cage cleaning. On PND21, the MS and control pups were weaned and then group-housed by sex (3–4 per cage). Since previous studies have shown that estrogen plays a key role in regulating neuronal activity and animal behavior and alterations in estrogen signaling are linked to a range of neurological and psychiatric conditions [25, 33], all subsequent experiments were carried out only on male offspring [27].

All procedures involving animals were approved and carried out according to Institutional Animals Care Committee of Renmin Hospital of Wuhan University guidelines.

### Experimental design

####  Experiment 1: the effects of MS on behavior, neuronal loss, M1 microglial activation, and pro-inflammatory cytokine, NAD^+^, Sirt3, and SOD2 expression in adolescent and adult rats

**Fig. 1:**
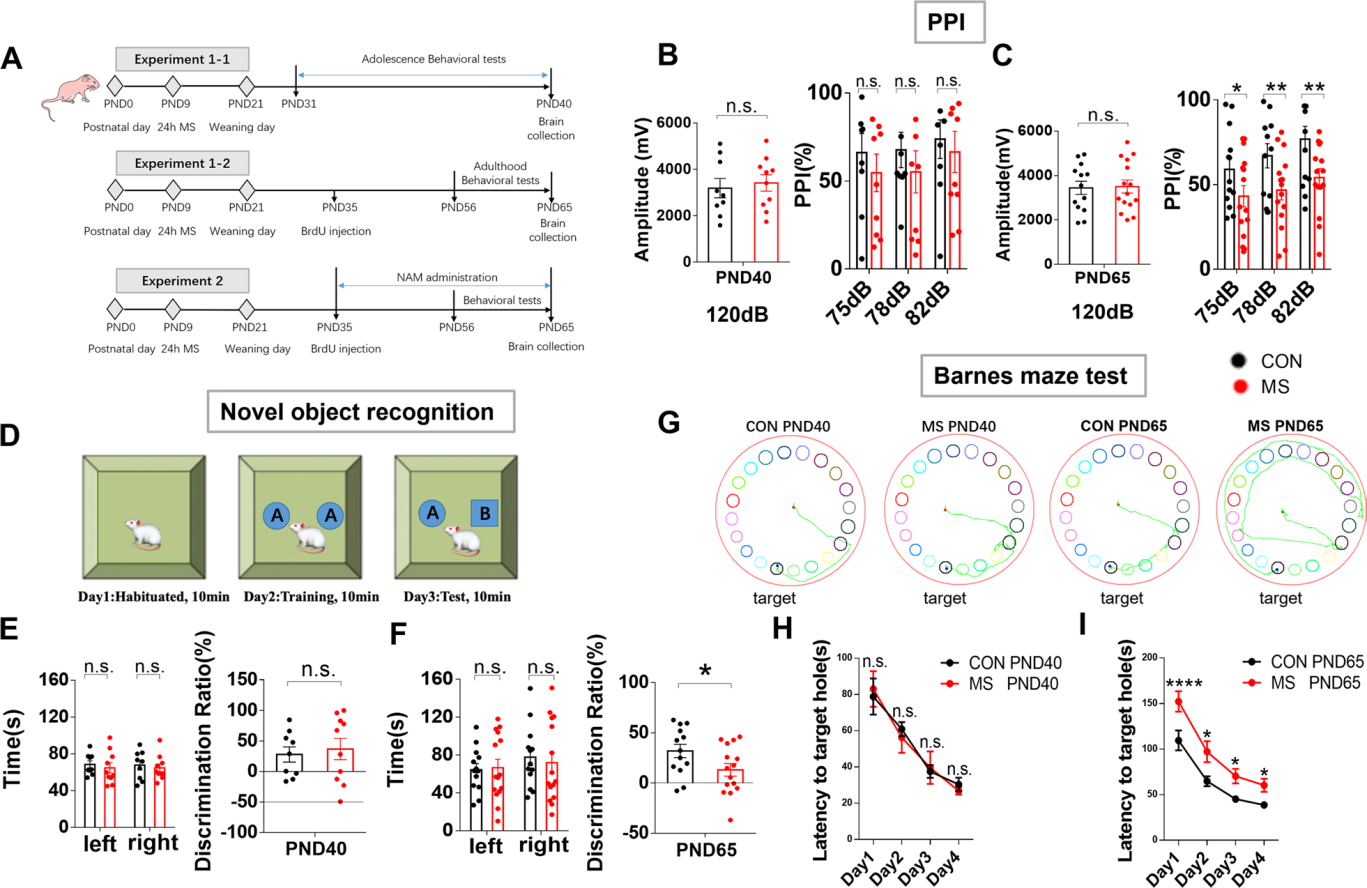
MS rats exhibit age-related impairments in sensorimotor gating and cognitive behaviors at PND65. **A** Experimental protocol. Litters were subjected to MS during lactation on PND9. Behavioral tests were performed during PND31-40 and PND56-65. Rats received BrdU injections intraperitoneally on PND35 and NAM from PND35 to PND65. PND, post-natal day; MS, maternal separation; BrdU, 5-bromo-2'-deoxyuridine. **B** The baseline startle response to an auditory-evoked startle stimulus of 120 dB is shown in the left panel. Percentage PPI of the auditory startle reflex for different pre-pulse intensities on PND40 in the right panel. **C** The baseline startle response to an auditory-evoked startle stimulus of 120 dB is shown in the left panel. Percentage PPI of the auditory startle reflex across different pre-pulse intensities on PND65 in the right panel. **D** Schematic of the novel object recognition experiment. **E**, **F** On day 2, time was spent exploring the two identical sample objects within the 10-min period. On day 3, time was spent exploring novel and old objects during the 10-min period on PND40 and PND65. **G** Trajectory graphs providing examples of the route taken to explore the target location by all groups in the Barnes maze test. The red and blue dots represent the start and end positions, respectively. The green line represents the trajectory. The target hole is labeled with a black circle. **H** The latency to the target hole in the Barnes maze test at PND40. **I** The latency to the target hole in the Barnes maze test at PND65. PND40 (CON, *n* = 9; MS, *n* = 10) and PND65 (CON, *n* = 13; MS, *n* = 15). *n.s.* not significant; *p < 0.05, **p < 0.01, and ***p < 0.001 as compared with controls. The data are represented as mean ± SEM

On PND9, dams and their pups were randomly divided into a control group and MS group (55 pups in each). Rats in the control group were not subjected to any treatment (normal controls), whereas rats in the MS group were exposed to MS as described above. During the adolescent (PND31–40) and adult stages (PND56–65), 15–20 pups were randomly selected from each group and subjected to behavioral testing. Animals were killed on PND40 and PND65 after behavioral testing, and there was no crossover between groups or carry-over of behavioral testing in the same group. The experimental flowchart is shown in Fig. 1A. The remaining pups were used in Experiment 2.

###  Experiment 2: the effects of administering NAM during adolescence on behaviors, neuronal loss, microglial activation, and pro-inflammatory cytokine, NAD^+^, Sirt3, and SOD2 expression in the hippocampus of adult MS rats

On PND10 after MS (or no treatment), each infant group was randomly divided into two subgroups (10–15 animals in each group). The resulting four groups (PND35) were: control and saline (Con + Saline), MS and saline (MS + Saline), control and NAM (Con + NAM), and MS and NAM (MS + NAM). The NAM groups were gavaged with NAM (100 mg/kg/day, diluted in saline; Sigma-Aldrich, St Louis, MO, USA; catalog no. N0636) for 30 days from PND35 to PND65. The NAM doses and treatment protocol were as previously [43, 55]. The saline groups received saline (1 ml/kg/day) from PND35 to PND65 as vehicle control. All rats were killed on PND65 after behavioral testing, and again there was no crossover between groups. The experimental flowchart is shown in Fig. 1A.

### Behavioral testing of offspring

Experimental rats underwent cognitive evaluation during adolescent (PND31–40) and adult (PND56–65) developmental stages. The novel object recognition test was completed over the first 3 days, while a standard behavioral protocol, the Barnes maze test, was completed between day four and day eight to evaluate cognition, including working and spatial recognition memory. On the last day, a pre-pulse inhibition (PPI) test was completed to measure sensorimotor gating. All behavioral tests were performed between 08:00 and 18:00. Each rat was assessed for each behavioral measure. After each trial, the apparatus was cleaned with 75% alcohol.

#### Novel object recognition test

The novel object recognition test was performed using the open-field apparatus and had three components: habituation, training, and testing [16]. Rats were placed in the center of the open field to adapt to the apparatus for 24 h. During the training experiment, the rats were allowed to explore two identical objects for 10 min. The test experiment started 24 h later. Rats were allowed to explore one familiar and one novel object (replacing a training object) for 10 min, with locomotion tracked by video and analyzed using Panlab SMART v3.0 (Barcelona, Spain). The formula for calculating preference for exploring the novel object was: [(time spent in the novel subject) / (total time spent in two subjects) × 100%] (Fig. 1D).

#### Barnes maze test

We examined spatial memory with the Barnes maze [34], which was elevated 140 cm above the floor and consisted of 20 holes located evenly on the surface periphery, each 10 cm in diameter. The target box was a hole that connected to a dark chamber, allowing the animal to escape from bright light exposure. The day before the formal experiment, animals were adapted to the target box for four minutes. On the first day, each animal was placed in the center black cube of the maze for five seconds and permitted to explore the maze to find the target box when the cube was removed. Once the animal entered the escape box, it was left there for 30 s; if it failed to find the target box within three minutes, it was taken to the target box and allowed to remain in the target box for one minute. Each animal underwent two trials during the day with an interval of four hours. The latency time to reach the target box was recorded. The test was performed over 4 days. The whole process was monitored by a digital camera and a computer system (Fig. 1G).

#### PPI testing

PPI testing was conducted in a sound-attenuated chamber equipped with a small Plexiglas cage mounted on a load-cell platform to digitize the pressure generated by the startled rat (AniLab Scientific Instruments Co., Ltd., China. www.anilab.cn. This test was performed with a broadband white noise of 70 dB. A high-pass (> 4 kHz) white noise was used as the pulse stimulus. Following acclimatization for 5 min, a sequence of five startle stimuli (120 dB of startle stimulus for 20 ms) was applied. During the testing phase, the initial delay was 50 ms followed by a 20-ms impulse stimulus (75, 78, or 82 dB), a 100-ms delay, followed by a 40-ms startle stimulus of 120 dB randomly applied for about 40 trials by software control. The interstimulus interval ranged from 10 to 30 s as shown in Additional file 1. The formula for calculating the percentage PPI caused by the intensity of each pre-pulse was [1-(startle amplitude on pre-pulse trial)/(startle amplitude on pulse alone)] × 100% [65].

### Sample collection

After completing behavioral testing on PND40 and PND65, the animals were killed using sodium thiopental (50 mg/kg, i.p.). Hippocampal tissue was taken from about ten animals in each group and frozen immediately in dry ice and then transferred to − 80 °C storage until required for further dissection and gene and protein expression analyses. Four animals in each group previously given a single intraperitoneal injection of BrdU (200 mg/kg in 0.9% sterile saline solution) on PND35 were perfused with 4% paraformaldehyde and their brains removed for immunofluorescence analysis.

### ***NAD***^+^***quantification***

NAD^+^ levels in the hippocampus were measured using NAD^+^ assay kits according to the manufacturer’s instructions (BioChain, Hayward, CA, USA). The method is based on the glucose dehydrogenase cycling reaction, in which tetrazolium dye 3-(4,5-dimethyl-2-thiazolyl)-2,5-diphenyltetrazolium bromide (MTT) is reduced by NADH in the presence of phenazine methosulfate. The product absorbance, measured at 565 nm, is proportional to the NAD^+^ concentration in the sample, calculated based on a standard curve.

### Protein extraction and western blot analysis

Total protein was extracted from hippocampal tissues with protein lysis buffer (Beyotime Biotech, China). The protein concentration was determined with a BCA kit (P0010S, Beyotime Biotech). The proteins in each sample were separated by 10% SDS–polyacrylamide gel electrophoresis and transferred onto polyvinylidene fluoride (PVDF) membranes. Western blot analysis was carried out using the following primary antibodies raised against target proteins: rabbit anti-acetyl lysine (dilution 1:1000, ab190479, Abcam, Cambridge, UK), rabbit anti-Sirt3 (dilution 1:1000, ab189860, Abcam), rabbit anti-SOD2 (dilution 1:1000, ab68155, Abcam), rabbit anti-acetyl-SOD2 (dilution 1:1000, AF3751, Affinity Biosciences, China), and rabbit anti-β-actin (dilution 1:1000, ab181602, Abcam) antibodies overnight at 4 °C. The following secondary horseradish peroxidase (HRP)-conjugated antibodies were used at 1:5000 dilution: goat anti-rabbit HRP (12–348, Millipore, Watford, UK) and goat anti-mouse HRP (sc-2005, Santa Cruz Biotechnology, Dallas, TX, UK). Blots were visualized with enhanced chemiluminescence detection reagents using a Chemidoc TM Touch Imaging System (Bio-Rad, Hercules, CA, USA).

### RNA preparation and gene expression analysis of pro-inflammatory cytokines by quantitative real-time polymerase chain reaction (qPCR)

For total RNA isolation and PCR, RNA was isolated with TRIZOL reagent (Invitrogen, Carlsbad, CA, USA) as recommended by the manufacturer, followed by chloroform extraction and isopropanol precipitation. The RNA was resuspended in nuclease-free water, and its concentration and purity were measured using a NanoDrop 2000 spectrophotometer (Thermo Fisher Scientific, Waltham, MA, USA). Then, total RNA (2 μg) was reverse transcribed into cDNA using a PrimeScript RT Kit (RR820A, Takara Bio Inc., Shiga, Japan). The reaction mixture was added to the RNA solution, and the RNA/reagent mixture was incubated at 42 °C for 1 h, further heated to 70 °C for 5 min, and then cooled to 48 °C. qRT-PCR was performed on a Bio-Rad Connect Real-Time PCR platform (Bio-Rad). PCRs were performed in a 20 μl volume with SYBR Master Mix with 1 μl pre-primer and 1 μl post-primer. PCR conditions were: 95 °C for 30 s, 40 cycles of 95 °C for 5 s and 60 °C for 30 s, 95 °C for 10 s, and 60 °C for 5 s. The 2^−ΔΔCT^ method was used to calculate mRNA expression levels using *Gapdh* as the reference gene. The qRT-PCR primer sequences are listed in Table 1.

**Table 1:** Specific primer sequences for target genes

Gene	Sense primer	Anti-sense primer
TNF-α	CTGGCGTGTTCATCCGTTCT	AGCCCATTGGAATCCTTGC
IL-1β	TGTGATGTTCCCATTAGAC	AATACCACTTGTTGGCTTA
IL-6	CCACTGCCTTCCCTACTT	TTGGTCCTTAGCCACTCC
GAPDH	GACATGCCGCCTGGAGAAAC	AGCCCAGGATGCCCTTTAGT

### Immunofluorescent staining assay

Rats were anesthetized and perfused through the heart initially with PBS and then with 4% paraformaldehyde. The brain was then post-fixed in 4% paraformaldehyde overnight. Brain tissues were embedded in paraffin and sectioned at 4 μm thickness. The paraffin-embedded tissue sections were dewaxed in xylol, rehydrated, placed in sodium citrate, microwaved for antigen retrieval, washed with PBS, and then blocked with 1% BSA (Roche, Basel, Switzerland) in PBS for 2 h at room temperature. Sections were incubated at 4 °C overnight with primary antibodies: rabbit anti-Sirt3 (1:100, ab189860, Abcam), goat anti-Iba1 (1:100, ab5076, Abcam), mouse anti-NeuN (1:100, ab104224, Abcam), goat anti-BrdU (1:100, ab1893, Abcam), or rabbit anti-c-fos (1:100, ab190289, Abcam) followed by incubation with an Alexa Fluor CY3-conjugated goat anti-rabbit IgG secondary antibody (1:250, Boster Biotech, China), an Alexa Fluor FITC-conjugated donkey anti-goat IgG secondary antibody (1:200, Boster Biotech, China), an Alexa Fluor FITC-conjugated goat anti-mouse IgG secondary antibody (1:250, Boster Biotech, China), an Alexa Fluor CY5-conjugated goat anti-rabbit IgG secondary antibody (1:200, Boster Biotech, China), or an Alex Fluor CY3-conjugated monkey anti-goat IgG secondary antibody (1:200, Boster Biotech, China) for 60 min. DAPI (Thermo Fisher Scientific) was used as a nuclear stain. Images were obtained under an inverted fluorescence microscope (IX53, Olympus, Tokyo, Japan). ImageJ (National Institutes of Health, Bethesda, MD) was used to analyze the integral optical density (IOD) of the target protein. Values obtained from three sections per animal were averaged for statistical analysis.

### TUNEL assay

TUNEL staining was performed on paraffin-embedded sections using the In Situ Cell Death Detection Kit, Fluorescein (ZSGB-Bio, China). This kit labels the DNA strand breaks generated during apoptosis, allowing the differentiation of apoptosis from necrosis. The procedure was carried out according to the manufacturer’s instructions. Slides were incubated for 30 min at 37 °C in 20 μg/ml proteinase K working solution, rinsed with PBS, and the area around the sample dried. The slides were then incubated with 50 μl of TUNEL reaction mixture containing terminal deoxynucleotidyl transferase (TdT) for 60 min in a dark, humidified atmosphere at 37 °C. After rinsing three times with PBS, slides were analyzed for positive cells.

### Data analysis and statistics

Data are presented as means ± standard error of the mean (SEM) and were analyzed with SPSS Statistics v20.0 (IBM Statistics, Armonk, NY, USA). Data from Experiment 1 were analyzed using an independent sample *t*-test. In Experiment 2, the data were analyzed by analysis of variance (ANOVA) to determine group differences. A p-value < 0.05 was regarded as significant.

## Results

### *The effects of MS on behavior, neuronal loss, M1 microglial activation, and pro-inflammatory cytokine, NAD* + *, Sirt3, and SOD2 expression in rats at PND40 and PND65*

#### MS rats exhibit abnormal PPI and cognitive dysfunction at PND65 but not PND40

To investigate the effect of MS on rat behavior, we conducted several behavioral tests. There were no differences between groups with respect to the baseline startle response to an auditory-evoked startle stimulus of 120 dB (Fig. 1B, left panel) in the PPI test, nor were there differences between groups on pre-pulse stimulus responses of 75 dB, 78 dB, and 82 dB (Fig. 1B, right panel). Furthermore, there were no significant differences in novel object recognition (Fig. 1E) and Barnes maze test (Fig. 1H) results between the MS groups and the control group at PND40.

At PND65, there were no differences between groups with respect to baseline startle responses to an auditory-evoked startle stimulus of 120 dB (Fig. 1C, left panel), and MS rats exhibited impaired PPI at varying pre-pulse intensities, indicating reduced inhibition of the startle stimulus compared with control rats (Fig. 1C, right panel). With respect to cognitive function using the novel object recognition test, MS and control rats took a similar amount of time to explore the two identical objects during the adaptation stage (Fig. 1F, left panel). However, the MS group spent relatively less time exploring the novel object when one of the objects was replaced after 24 h (Fig. 1G, right panel). In the Barnes maze test, MS rats showed deficiencies in spatial learning and memory at PND65. Compared with the controls, MS rats showed a greater latency to escape from day 1 to day 4 (Fig. 1I).

#### Neuronal loss in the hippocampus in MS rats

**Fig. 2:**
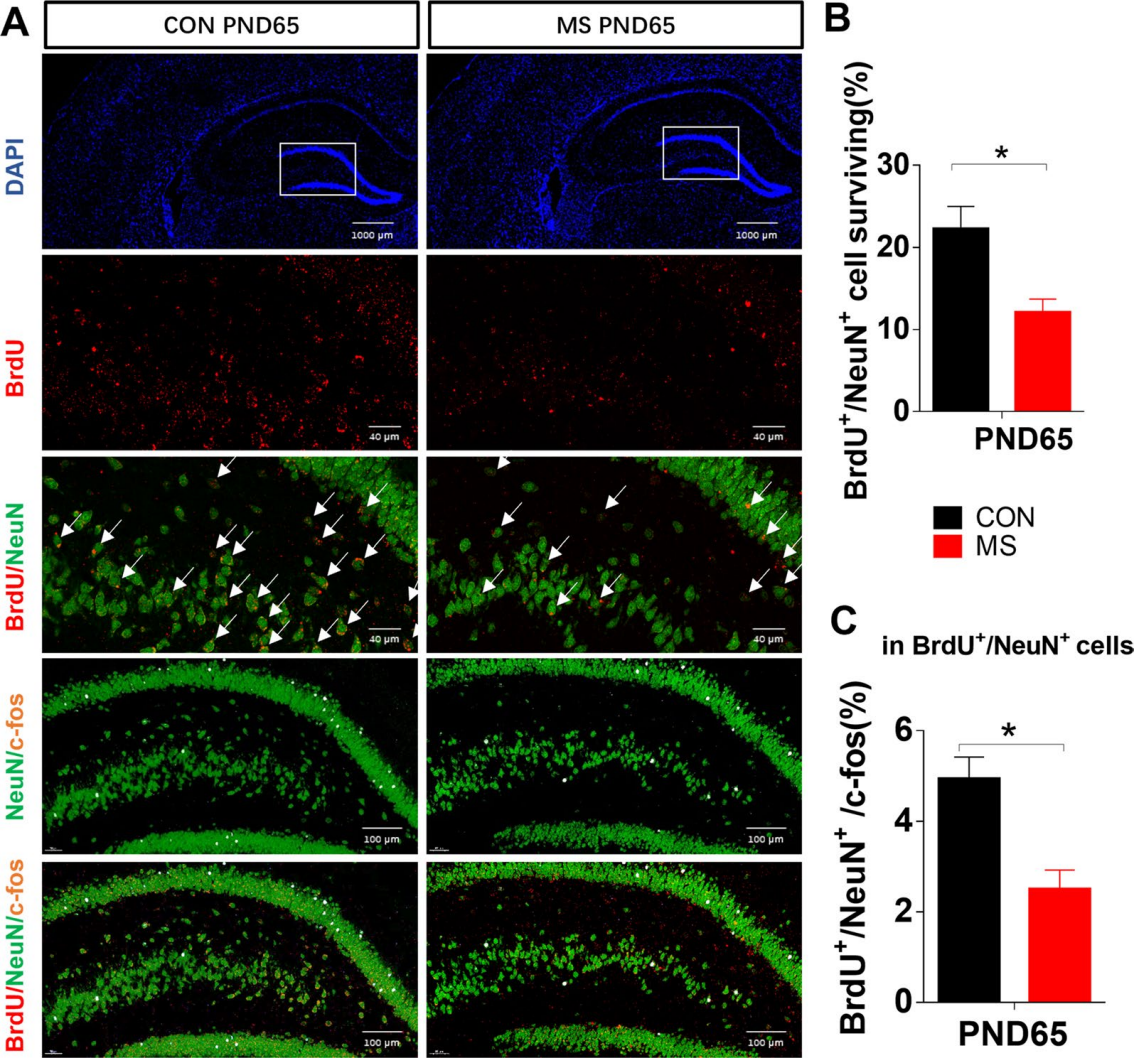
The survival and activity of puberty-born neurons. **A** Immunofluorescence was used to characterize the survival of puberty-born neurons. The BrdU/NeuN assay shows the survival of puberty-born neurons in the DG until PND65; the BrdU/NeuN/c-fos assay shows the active puberty-born neurons. NeuN (green pixels), BrdU (red pixels), c-fos (pink pixels), and DAPI (blue pixels). **B** The percentage of puberty-born (BrdU/NeuN) neurons in the DG. **C** The percentage of active puberty-born neurons (BrdU/NeuN/c-fos) in BrdU/NeuN cells. *P* values were calculated using Student’s *t*-test. Data are presented as mean ± SEM for each group (*n* = 4 per group). *p < 0.05

Two groups of rats were administered a single intraperitoneal injection of BrdU during adolescence to label the number of new neurons surviving from adolescence to adulthood. The survival of puberty-born neurons was reduced in the DG in MS animals, as measured by the BrdU:NeuN ratio (Fig. 2A, B). MS reduced the number of active neurons generated during puberty in the entire DG as measured by BrdU/NeuN/c-fos staining (Fig. 2A, C).

**Fig. 3:**
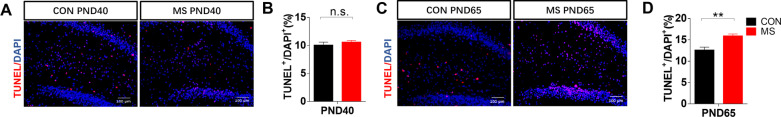
Neuronal apoptosis in the hippocampal dentate gyrus by TUNEL. **A**, **B** Neuronal apoptosis in the DG at PND40 was assessed by TUNEL staining. **C**, **D** Neuronal apoptosis in the DG at PND65 was assessed by TUNEL staining. Original magnification × 200. Data are presented as mean ± SEM for each group (*n* = 4 per group). *P* values were calculated using Student’s *t*-test. *n.s.* not significant; **p < 0.01

Neuronal apoptosis was assessed by TUNEL staining. There was no difference between groups at PND40 (Fig. 3A, B), while at PND65 the number of TUNEL‐positive cells increased in the MS group in the DG compared with the control group (Fig. 3C, D).

#### ***Depletion of NAD***^+^***and Sirt3 and increased protein acetylation, microglial pro-inflammatory responses, and cytokine expression in the hippocampus of MS rats***

**Fig. 4:**
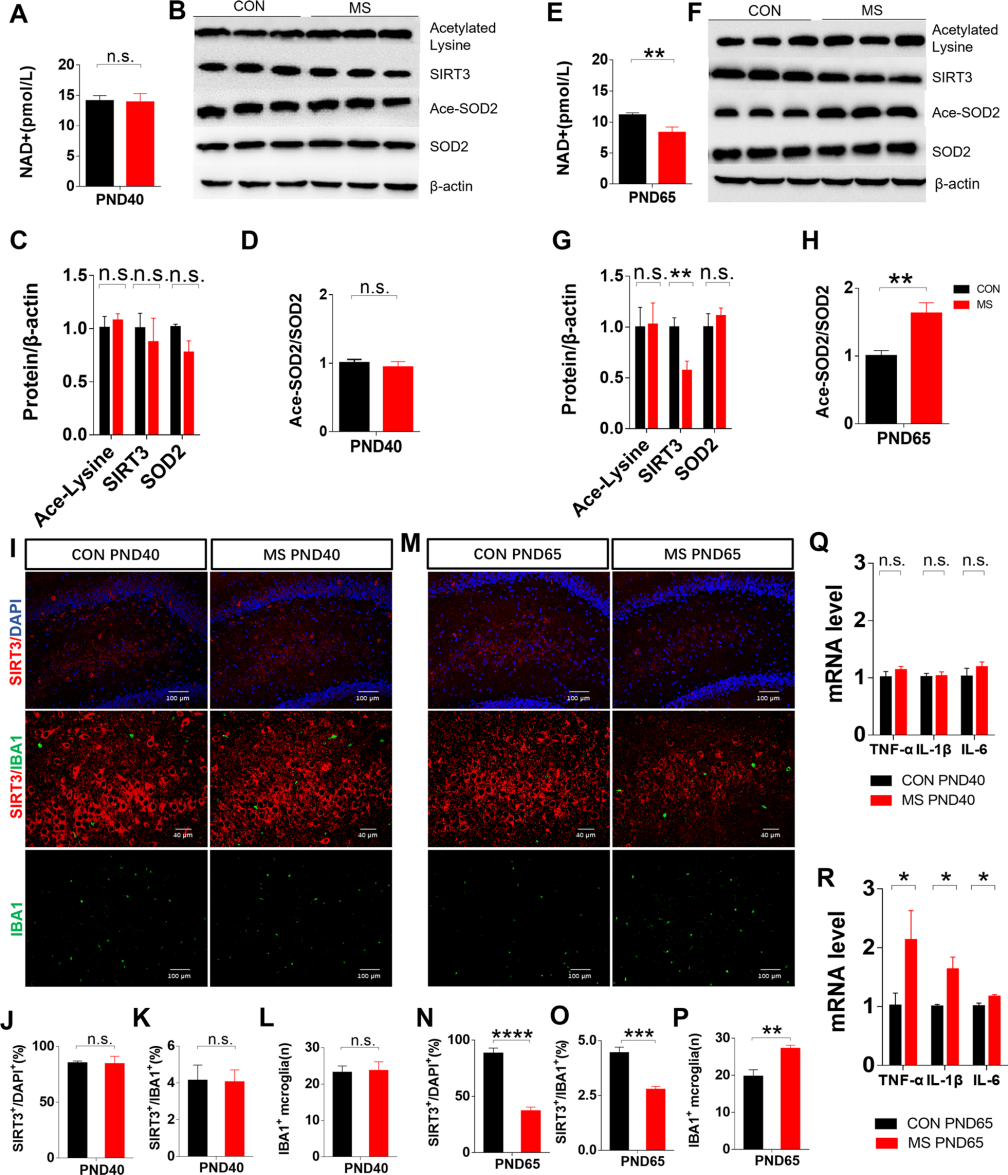
The expression of NAD^+^, Sirt3, and acetylated SOD2 and microglial inflammation. **A** NAD^+^ levels in the hippocampus at PND40 (CON, *n* = 8; MS, *n* = 8). **B**–**D** Immunoblots and quantification of Sirt3, acetylated SOD2, and total SOD2 in the hippocampus at PND40 (CON, *n* = 4; MS, *n* = 4). (E) NAD^+^ levels in the hippocampus at PND65 (CON, *n* = 8; MS, *n* = 8). **F**–**H** Immunoblots and quantification of Sirt3, acetylated SOD2, and total SOD2 in the hippocampus at PND65 (CON, *n* = 6; MS, *n* = 6). **I** Representative immunofluorescence images show the expression of Iba1 (green pixels) and Sirt3 (red pixels) in the DG at PND40 (*n* = 4 per group). **J** Quantitative analyses of the percentage of Sirt3 expression. (K) Quantitative analyses of the percentage of Iba1 and Sirt3 co-labeling. **L** Quantitative analyses of Iba1^+^ immunostained cells. **M** Representative immunofluorescence images showing the expression of Iba1 (green pixels) and Sirt3 (red pixels) in the DG at PND65 (*n* = 4 per group). **N** Quantitative analyses of the percentage of Sirt3 expression. **O** Quantitative analyses of the percentage of Iba1 and Sirt3 co-labeling. (P) Quantitative analyses of Iba1^+^ immunostained cells. **Q** Real-time qPCR analysis of TNF-α, IL-1β, and IL-6 in the hippocampus at PND40 (CON, *n* = 4; MS, *n* = 4). **R** Real-time qPCR analysis of TNF-α, IL-1β, and IL-6 in the hippocampus at PND65 (CON, *n* = 6; MS, *n* = 6). Data are presented as mean ± SEM for each group. *n.s.* not significant; *p < 0.05, **p < 0.01, and ***p < 0.001.

We next measured the concentration of NAD^+^ and the expression of Sirt3 and SOD2 at PND40 and PND65. While there were no differences in these parameters at PND40, NAD^+^ and Sirt3 levels decreased significantly in the hippocampus of PND65 MS rats compared with controls. Although total SOD2 expression was unchanged, at PND65 there was an ~ 30% increase in SOD2 acetylation (Fig. 4E–H). Figure 4M–O shows that the proportion of Sirt3/Iba1 co-labeled areas decreased significantly in MS rats, while Figs. 4M and 4P show that the number of Iba1-immunostained cells significantly increased in the DG of MS rats. Furthermore, there was a significant increase in IL-1β, TNF-α, and IL-6 expression in the hippocampus of MS rats at PND65 (Fig. 4Q–R).

###  The effects of NAM administration during adolescence on behaviors, neuronal loss, microglial activation, and pro-inflammatory cytokine, NAD^+^, Sirt3, and SOD2 expression in the hippocampus of adult MS rats

#### Changes in behavior and cognitive function

**Fig. 5:**
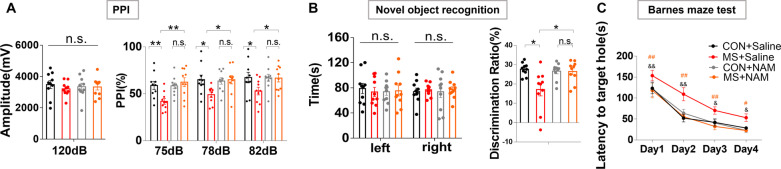
NAM administration attenuates PPI and cognitive deficits induced by MS. **A** The baseline startle response to an auditory-evoked startle stimulus of 120 dB is shown in the left panel. Effects of NAM administration (100 mg/kg) on PPI at different pre-pulse intensities in the right panel. **B** Effects of NAM administration on the novel object recognition assay in different groups. **C** Effects of NAM administration on the latency to the target hole in the Barnes maze test. ^&^*p* < 0.05, ^&&^*p* < 0.01 as compared to MS + Saline rats, ^#^*p* < 0.05, ^##^*p* < 0.05 as compared to MS + NAM rats. CON + Saline, *n* = 10; MS + Saline, *n* = 10; CON + NAM, *n* = 10; MS + NAM, *n* = 10. *n.s.* not significant; *p < 0.05 and **p < 0.01 as compared to controls. Data are represented as mean ± SEM

There were no differences between the four groups with respect to baseline startle response to an auditory-evoked startle stimulus of 120 dB (Fig. 5A, left panel). The impaired pre-pulse stimulus responses of 75 dB, 78 dB and 82 dB caused by MS were partially ameliorated after administration of 100 mg/kg NAM from PND35 to PND65 (Fig. 5A, right panel). With respect to the novel object recognition test, the MS + NAM group spent more time sniffing the novel object after 24 h than the MS + Saline group (Fig. 5B, right panel). In addition, the MS + NAM group had decreased latency entering the target hole compared with the MS + Saline group in the Barnes maze test (Fig. 5C, D).

#### The expressions of NAD+, Sirt3, SOD2, microglial pro-inflammatory responses, and pro-inflammatory cytokine expression in the hippocampus

**Fig. 6:**
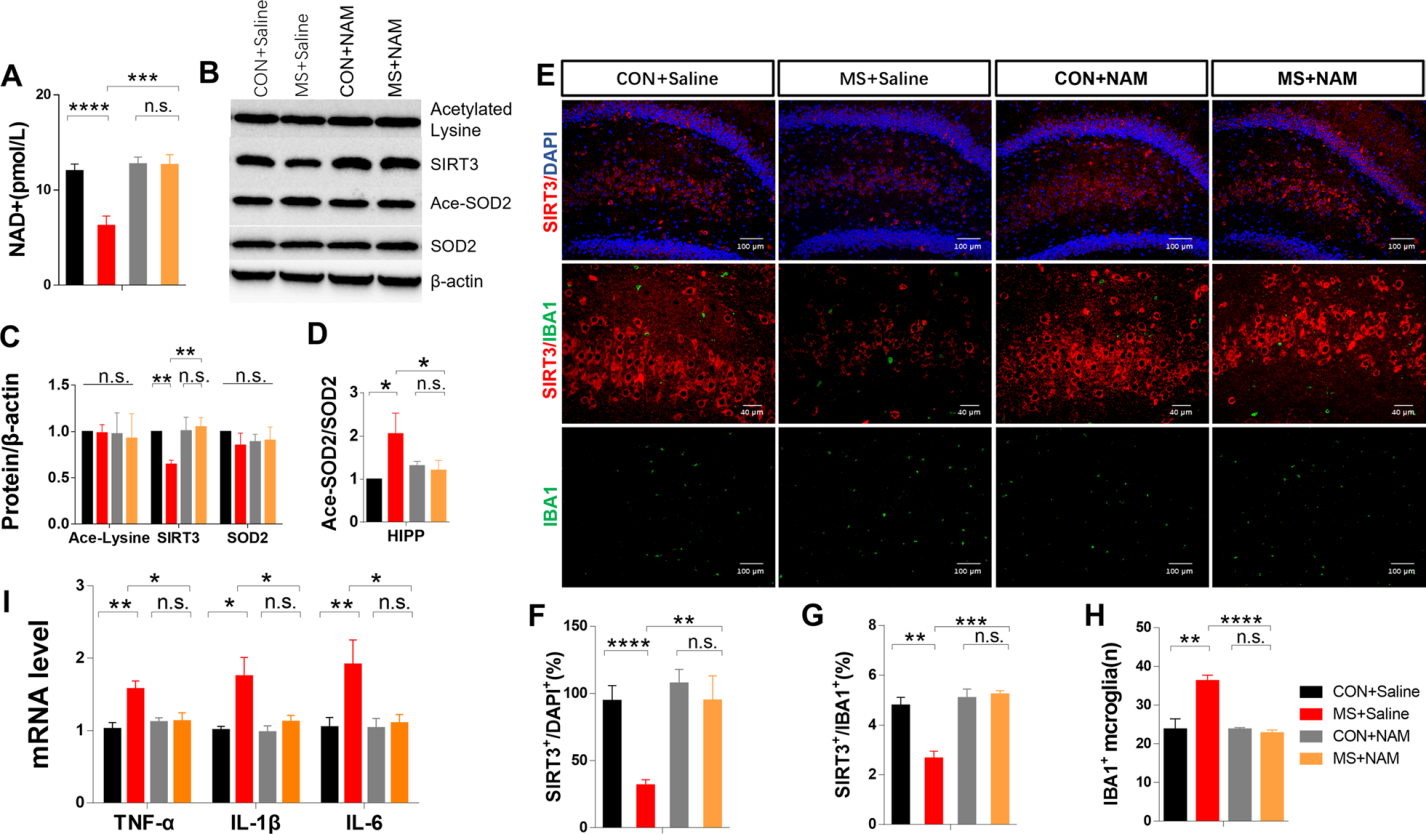
NAM administration reverses the effects of MS on NAD^+^, Sirt3, protein acetylation, and microglial inflammation. **A** NAD^+^ levels in the hippocampus (CON + Saline, n = 8; MS + Saline, *n* = 8; CON + NAM, *n* = 8; MS + NAM, *n* = 8). **B**—**D** Immunoblots and quantification of Sirt3, acetylated SOD2, and total SOD2 in the hippocampus (*n* = 6, per group). **E** Representative immunofluorescence images show the expression of Iba1 (green pixels) and Sirt3 (red pixels) in the DG (*n* = 4 per group). **F** Quantitative analyses of the percentage of Sirt3 expression. **J** Quantitative analyses of the percentage of Iba1 and Sirt3 co-labeling. **H** Quantitative analyses of the Iba1 + immunostained cells. **I** Real-time qPCR analysis of TNF-α, IL-1β, and IL-6 in the hippocampus (*n* = 6 per group). Data are presented as mean ± SEM for each group. *p < 0.05, **p < 0.01, and ***p < 0.001

Figure 6A shows the recovery of NAD^+^ in the hippocampus of MS rats after NAM administration. Compared with the MS group, there was increased hippocampal Sirt3 expression and decreased SOD2 acetylation in the MS + NAM group (Fig. 6B–D). Figure 6E, F shows that the proportion of Sirt3/Iba1 co-labeled cells increased in MS + NAM rats. The number of Iba1-positive cells decreased in the DG of MS + NAM rats (Fig. 6E and H), while IL-1β, TNF-α, and IL-6 expression decreased in the hippocampus in MS + NAM rats at PND65 (Fig. 6I).

#### Neuronal loss in the hippocampus

**Fig. 7:**
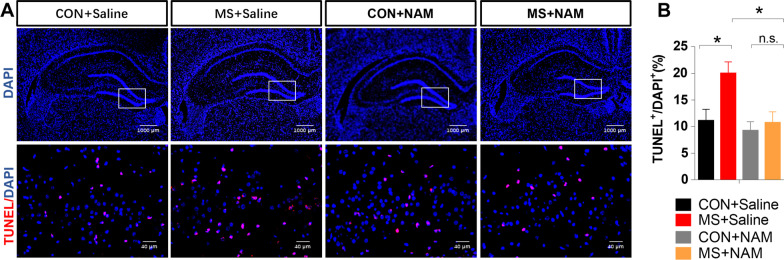
NAM administration reduces neuronal apoptosis in the DG. **A**, **B** Neuronal apoptosis in the DG was assessed by TUNEL staining at PND65. Data are presented as mean ± SEM for each group (*n* = 4 per group). *n.s.* not significant; *p < 0.05

**Fig. 8:**
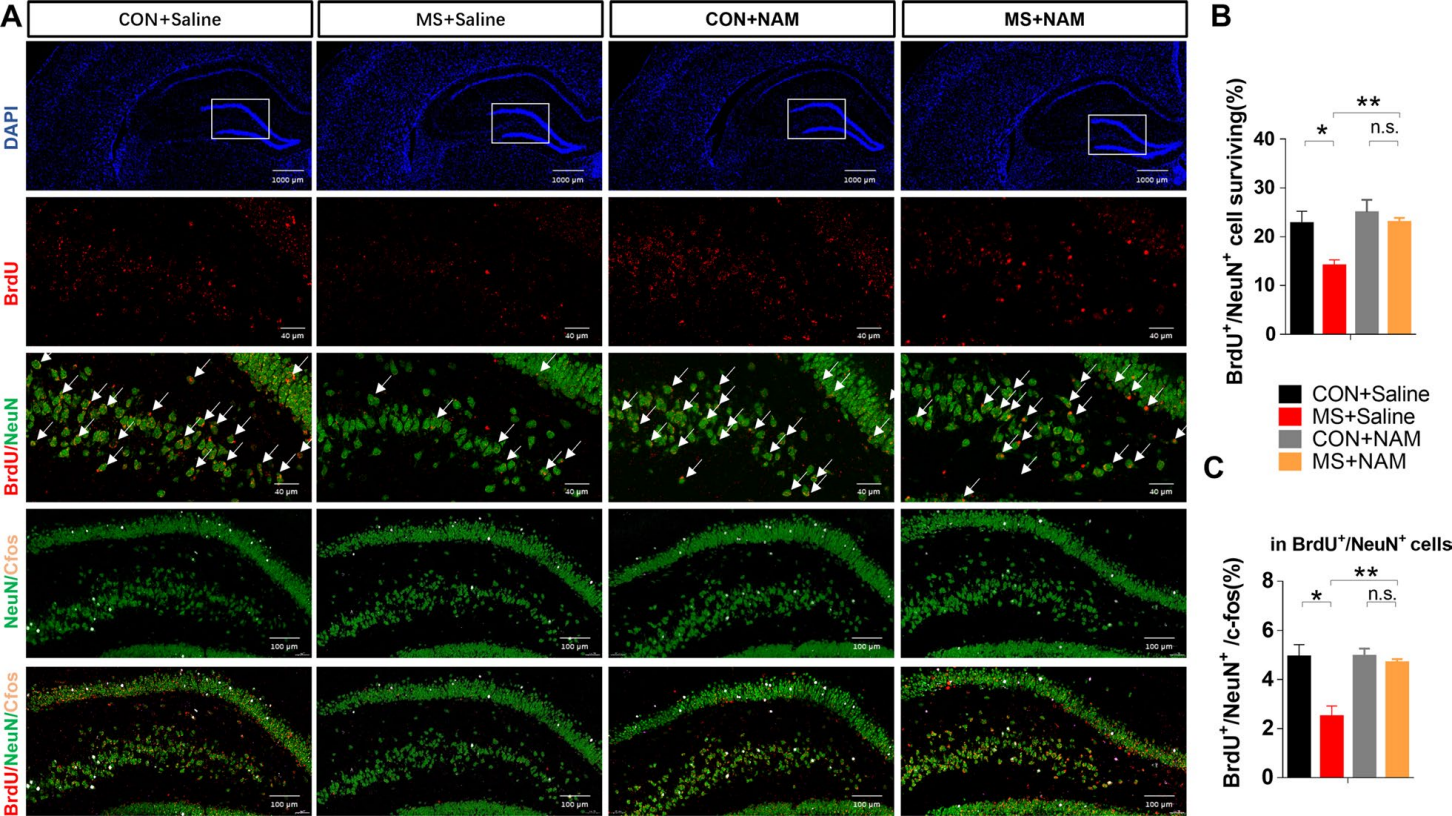
NAM administration recovers the survival and function of puberty-born neurons. **A** BrdU/NeuN assay shows the survival of puberty-born neurons in the DG until PND65; BrdU/NeuN/c-fos assay shows the active puberty-born neurons. NeuN (green pixels), BrdU (red pixels), c-fos (pink pixels), and DAPI (blue pixels). **B** The percentage of puberty-born neurons in DG neurons. **C** The percentage of active puberty-born neurons in BrdU/NeuN cells. Data are presented as mean ± SEM for each group (*n* = 4 per group). *n.s.* not significant; *p < 0.05, and **p < 0.01

**Fig. 9:**
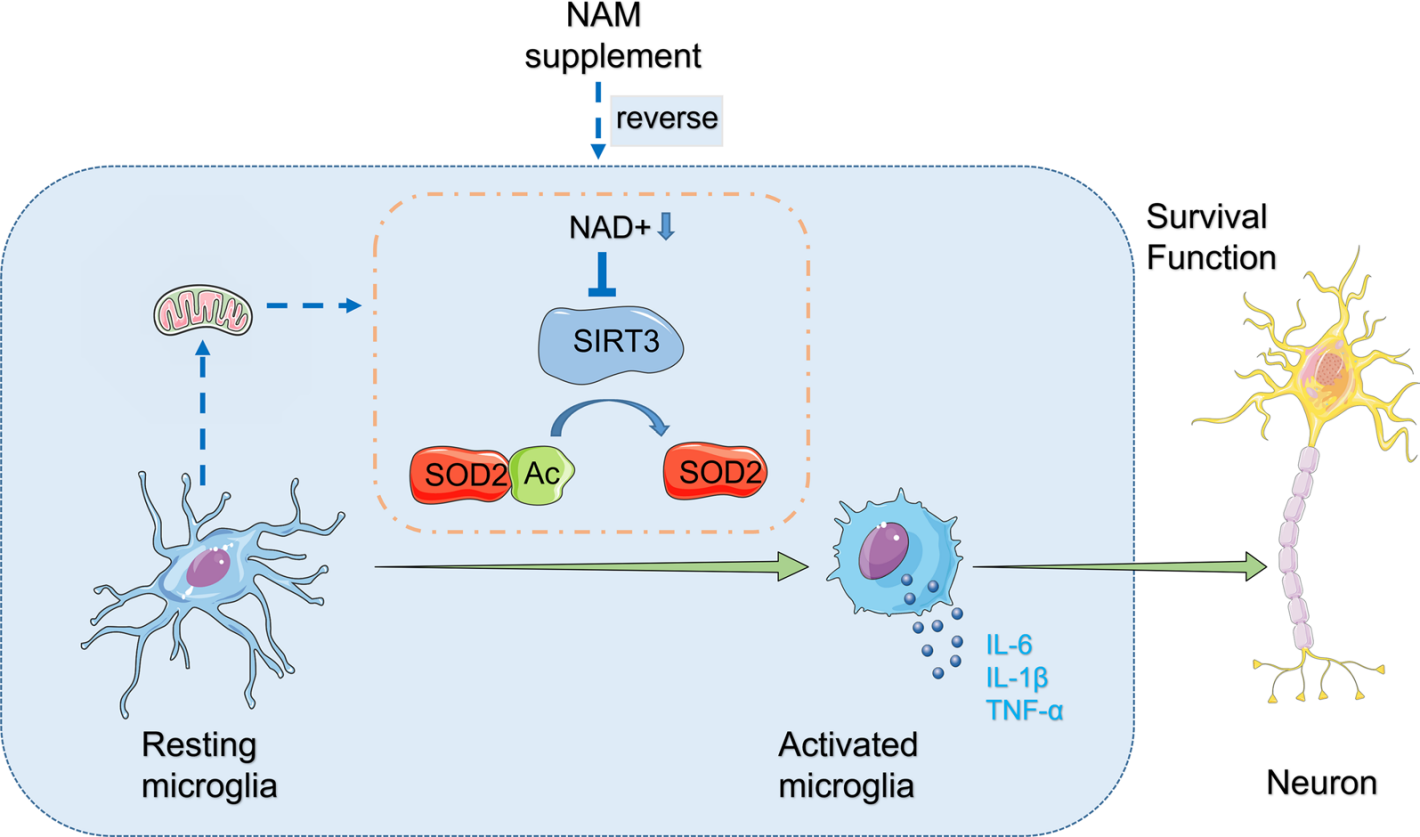
A proposed schematic illustrating how NAM administration might attenuate cognitive impairment in schizophrenia through the NAD^+^/Sirt3 pathway. NAD^+^ deficits in brain tissue downregulate Sirt3 expression in microglia, followed by increased protein acetylation and redox abnormalities. Microglia are activated and neuroinflammation is enhanced, which would induce neuronal damage and result in cognitive deficits. NAM administration can reverse these changes

NAM administration in the MS + NAM group decreased neuronal apoptosis, as assessed by TUNEL staining, compared with the MS + Saline group (Fig. 7A, B). MS reduced the survival of puberty-born neurons in the DG, as measured by BrdU/NeuN, which was abolished by NAM administration (Fig. 8A, B). NAM administration significantly increased the proportion of active neurons generated in adulthood in the MS group, measured by dividing the number of cells co-labeled with BrdU/NeuN by those triple-labeled with BrdU/NeuN/c-fos (Figs. 8A, C and 9).

## Discussion

There is now a growing body of evidence on the neurodevelopmental origins of schizophrenia, in which symptoms typically emerge during late adolescence or young adulthood [29]. Following MS on PND9, we compared behaviors in rats at PD40 and PD65, which correspond to adolescence and adulthood and are likely to reflect pre-symptomatic and symptomatic periods of development in schizophrenia. We found that MS rats on PND65 spent less time exploring novel objects in the novel object recognition test and had a greater latency to escape in the Barnes maze test. Additionally, PPI, which measures sensorimotor gating after the startle response, was also disrupted. Although the underlying neural mechanisms remain elusive, PPI impairments are present in several neuropsychiatric disorders, including schizophrenia [40, 58], indicating that PPI suppression is a translatable finding when assessing animal models of schizophrenia [63, 69]. It is interesting that these behavioral deficits were present on PND65 and not PND40, indicating that MS can lead to a significant decrease in cognitive function, including impaired remote memory and recognition memory. This is consistent with other studies [23, 53, 60], and indeed age-related abnormal behavioral phenotypes have also been reported in other animal models of schizophrenia such as those derived from poly (I:C)-induced gestational inflammation [28].

One question is why early adverse life experiences in this model do not show their effects until adulthood. Adolescence is a sensitive period of brain development. Perinatal stress is known to modulate the progression of pubescent brain development. There is evidence that neurons generated during the adolescent stage are involved in emotional regulation and hippocampal-dependent behaviors including trace and contextual fear conditioning, spatial navigation, pattern separation, and cognitive flexibility [3, 35, 62]. Given the important roles played by these neurons born in puberty, we administered a single intraperitoneal injection of BrdU on PND35 to label neuronal neogenesis at this age and track neuronal survival through adolescence into adulthood. We found that the survival of these mature puberty-born neurons was reduced in the hippocampus in MS rats on PND65. We further confirmed a significant increase in neuronal apoptosis in the DG in these adult animals through TUNEL staining, while there was no difference in adolescence. Puberty-generated cells differentiate into neurons and glia and are functionally incorporated into neural circuits that subserve behaviors [46]. The juvenile brain is more plastic than the adult brain, and puberty-generated neurons are especially responsive to learning, which can have significant consequences on the functional integrity of the hippocampus [18]. Thus, mature puberty-born neurons are functionally relevant for behavior, and ablation of this neuronal population might result in abnormal behaviors.

We found that microglia were overactivated and there was release of pro-inflammatory factors in MS rats on PND65 but not PND40. ELS is known to induce a persistent pre-activated state in microglia for years, and primed microglia have an exaggerated response to future life challenges such as stress, infection, and changes in hormone levels [12, 47]. The levels of several hormones change significantly during puberty, and previous studies have suggested that androgens promote microglial activation and phagocytic capacity [67]. This might partly explain our results. Microglia are the main effectors of inflammation in the CNS, responding to even small changes in the CNS. The view that hyperactivated microglial-mediated neuroinflammation contributes to neurotoxicity in the developing brain is supported by both in vitro and in vivo studies [8, 15, 68]. Therefore, the reduced survival of newborn neurons in the MS group on PND65 may be caused by over-activated microglia induced by ELS. Avoiding or reducing neuronal loss induced by microglial inflammation is therefore a potential mechanism to prevent the development of disease during the asymptomatic period of schizophrenia.

Previous studies have suggested that NAM may control inflammatory diseases by increasing NAD^+^ [64]. A primary purpose of our study was to explore whether NAM, as a precursor of NAD^+^, could prevent or reduce ELS-induced adverse changes in the CNS. We provide new evidence demonstrating the beneficial effects of NAM supplementation during adolescence, which appears to effectively inhibit microglial inflammation and avoid both ELS-induced reduction in puberty-born neurons and the consequent cognitive impairment. Our results are consistent with studies in adult animals indicating that NAM treatment attenuates cortical loss following closed-cortical injury [4, 30–32]. However, to our knowledge, this is the first study demonstrating that NAM acts as a neuroprotective agent that can be used during puberty to prevent neuronal loss. Administering NAM could be a relatively simple and safe way to prevent neuronal loss and improve hippocampal function in individuals who have had ELS.

We also investigated the molecular mechanisms by which NAM might reduce microglial inflammation in MS. NAM reversed MS-induced deficits in NAD^+^. NAD^+^ is a fundamental mediator of antioxidant defenses, and mitochondrial NAD^+^ degradation would therefore reduce antioxidant capacity [38]. The brain is particularly vulnerable to oxidative stress due to its high content of highly oxidizable polyunsaturated fatty acids and the relatively low antioxidant load compared with other tissues [5, 22]. Evidence from proteomic and metabolomic studies has suggested that oxidative stress is elevated in schizophrenia, with disturbed mitochondrial function and reduced antioxidant capacity [39]. A recent clinical study showed redox dysregulation in first-episode schizophrenia patients demonstrated by a decreased NAD^+^/NADH ratio in the prefrontal cortex measured by ^31^P magnetic resonance spectroscopy [37]. Reduced antioxidant capacity in the brain could result in neuroinflammation and affect neuronal functions such as neurotransmitter synthesis and synaptic plasticity [48]. Sirt3 is a mitochondrial deacetylase, a class of proteins involved in aging and stress damage, and it is NAD^+^ dependent [21]. Sirt3 plays important roles in the inhibition of neuroinflammation and protecting blood–brain barrier integrity in ischemic stroke [71]. In our research, NAD^+^ and Sirt3 levels were decreased in the brains of MS rats in adulthood (PND65), along with increased acetylated SOD2. SOD2 plays a key role in mitochondrial antioxidant defenses, and increased SOD2 acetylation can decrease antioxidant function [44, 52, 73]. It was worth noting that we detected the overall protein acetylation level in the hippocampus of animals and found that there was no difference between MS and control rats on PND40 or PND65, indicating that Sirt3 decreases might play an important role in the increase in acetylated SOD2. To confirm the importance of mitochondrial antioxidant dysregulation in pro-inflammatory responses in microglia, we examined co-expression of Iba1 and Sirt3 and found that Sirt3 expression decreased significantly in microglia the MS group on PND65 but not on PND40. The number of microglia significantly increased, accompanied by pro-inflammatory cytokine (TNF-α, IL-1β, IL-6) upregulation in the MS group at the adult stage. NAM administration reversed or attenuated all these consequences of MS.

The findings of the present study suggest that NAM administration may act by upregulating NAD^+^ and Sirt3 expression to increase the antioxidant activity of SOD2 and thereby reduce microglial inflammation and neuronal loss, at least in the MS model. However, we did not explore whether it can inhibit microglial inflammation and improve cognitive impairment in model animals by regulating Sirt3 expression. Further investigations are now required to fully elucidate the mechanisms behind this association. Furthermore, we only explored changes at PND40 and PND65, so could not identify the exact point when immunity was activated. Additionally, we only administered NAM to model rats during adolescence and not in adulthood, so we do not know whether NAM has an effect on emerging or established abnormal schizophrenia-like behaviors. Finally, we acknowledge that an important limitation of this work is the use solely of male animals. While this was deliberate to eliminate the added variability that can be caused by fluctuating estrogen levels, future work will need to address the effects in females and possible sex differences.

## Conclusions

This study shows that exposure to ELS may result in age-dependent cognitive impairment, concomitant with an ongoing decrease in survival and activity of puberty-born neurons in the hippocampus. NAM administration in adolescence rescued behavioral performance and restored the survival and activity of puberty-born neurons to normal levels. Our study provides new information about the etiology of schizophrenia. NAD^+^ supplementation may provide a novel and safe preventative strategy for individuals who have undergone childhood trauma and are at higher risk of developing psychiatric disorders such as schizophrenia.

## Supplementary Information

**Additional file 1.** Line graph depicting the stimulus protocol to assess the sensorimotor gating.

## Data Availability

The datasets used and/or analyzed during the current study are available from the corresponding author on reasonable request.

## Abbreviations

ELS: Early life stress
NAM: Nicotinamide
MS: Maternal separation
PND: Postnatal day
PPI: Pre-pulse inhibition
NAD^+^: Nicotinamide adenine dinucleotide
Sirt3: Sirtuin3
SOD2: Superoxide dismutase 2
NAM: Nicotinamide
DG: Dentate granule
CNS: Central nervous system
