# Supplementary figures and images for: Nicotinamide reverses deficits in puberty-born neurons and cognitive function after maternal separation
Source: J Neuroinflammation. 2022 Sep 21;19:232. doi: 10.1186/s12974-022-02591-y (PMC9494869; doi:10.1186/s12974-022-02591-y)

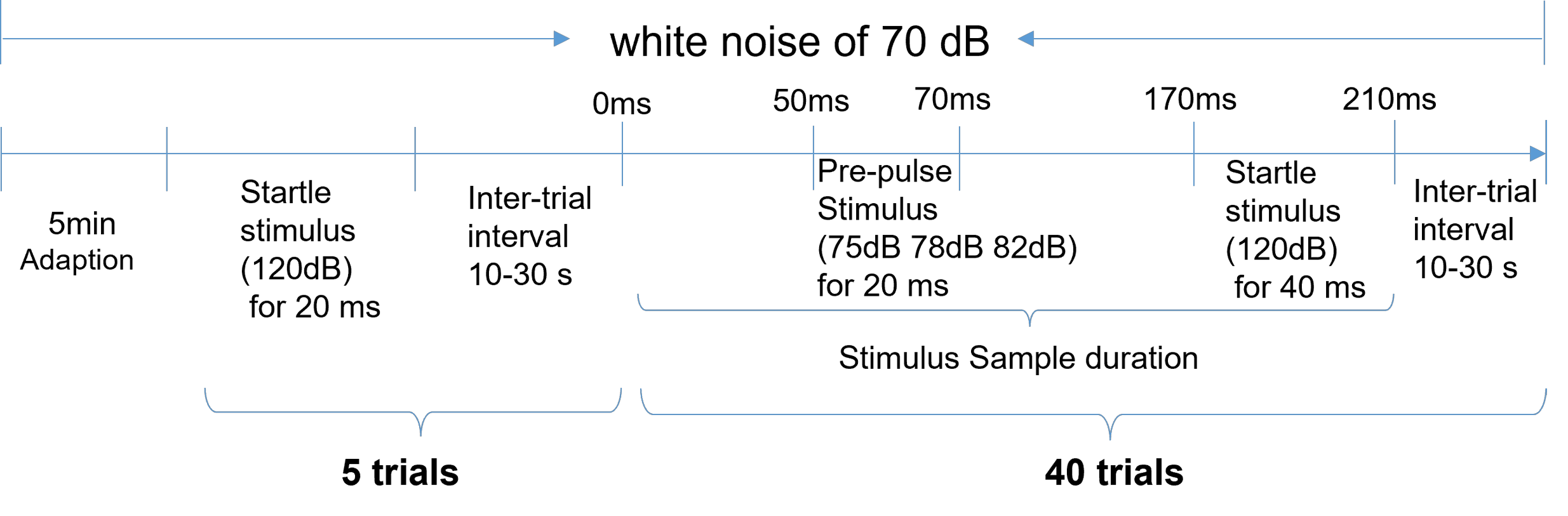

Supplement: Supplementary file 1 — Additional file 1. Line graph depicting the stimulus protocol to assess the sensorimotor gating. [file 12974_2022_2591_MOESM1_ESM.zip › Supplementary.tif]
